# Chaulmoogra Oil in Leprosy

**Published:** 1916-05

**Authors:** 


**Chaulmoogra Oil in Leprosy.** Vahram, Le Prog. Med., Feb. 5, reports two cases, receiving about 30 injections each. A pseudo-celloid solution is employed, containing 72/100 m.g. of chaulmoogra oil per C.C. The initial dose is 1/4 C.C., increased by 1/10 C.C. up to 2 C.C. and holding at this dose till 20 injections have been given intravenously. Then begin hypodermic injections at 1/2 C.C., increasing by 1/2 or 1 C.C. up to 5 C.C. and holding at this amount to the 20th injection. Injections of both forms are given every second day. Good results, cicatrization, etc., increase in red cells, to normal, absence of haemolysis or of modification of respiration or urinary secretion.

